# New perspectives on the nutritional factors influencing growth rate of *Candida albicans* in diabetics. An *in vitro* study

**DOI:** 10.1590/0074-02760170098

**Published:** 2017-09

**Authors:** Adrian Man, Cristina Nicoleta Ciurea, Dan Pasaroiu, Ana-Ioana Savin, Felicia Toma, Floredana Sular, Luigi Santacroce, Anca Mare

**Affiliations:** 1 University of Medicine and Pharmacy, Department of Microbiology, Tîrgu Mures, Romania; 2 University of Medicine and Pharmacy, Tîrgu Mures, Romania; 3 University of Medicine and Pharmacy, Department of Laboratory Medicine, Tîrgu Mures, Romania; 4 Emergency Clinical County Hospital, Tîrgu Mures, Romania; 5 University of Bari Aldo Moro, Policlinico University Hospital, Ionian Department and Microbiology and Virology Service, Bari, Italy

**Keywords:** Candida albicans, diabetes, yeast infections, fructose, flow-cytometry, glycemic control

## Abstract

**BACKGROUND:**

The link between *Candida albicans* and diabetes mellitus is well-acknowledged, but incompletely elucidated.

**OBJECTIVES:**

The purpose of this study is to assess the growth rate of *C. albicans* (CA) in the presence of different concentrations of glucose and fructose, two of the main pathophysiologic and nutritionally relevant sugars in diabetic patients, in order to obtain a better understanding of the nutrient acquisition strategy and its possible relation to the hyperglycemic status of diabetic patients.

**METHODS:**

The effects of different concentrations of glucose and fructose (1000 mg%, 500 mg%, 250 mg% and 100 mg% w/v) on the growth rate of CA have been studied by flow-cytometry.

**FINDINGS:**

We found that glucose concentration is directly related to CA growth, which may be linked to the frequent yeast infections that occur in non-controlled diabetic patients; we also show that fructose inhibits CA growth rate.

**MAIN CONCLUSIONS:**

As a consequence of our hypothesis, the study demonstrates that fructose-containing food may prevent the development of candidiasis, at least in oral sites.

It is well known that diabetes mellitus is one of the most prevalent chronic conditions throughout the world, with an estimation of 56 million cases, a prevalence of 8.5% in Europe in 2013 and rising (Tamayo et al. 2014). The high morbidity is mostly related to chronic macrovascular and microvascular complications such as nephropathy, neuropathy, retinopathy, heart complications, related obesity, but also infectious complications, due to the hyperglycemic environment (Casqueiro et al. 2012, Tamayo et al. 2014, Gregg et al. 2016). Yeast infections in diabetic patients are common, candidiasis being described as part of the major infectious pathology: oral and esophageal candidiasis, pyelonephritis or cystitis (Casqueiro et al. 2012). In addition, recent reviews have focused on the relationships between oral microbial flora, oral hygiene and glycemic control in diabetics (Santacroce et al. 2010, Monea et al. 2017).

Although the link between *Candida albicans* (CA) and diabetes mellitus is well-recognised, it is still incompletely elucidated (Soysa et al. 2006). Oral candidiasis is frequently caused by *albicans* species and presents many clinical forms (pseudomembranous candidiasis, *Candida-*related leukoplakia, angular cheilitis, glossitis or stomatitis). *Candida* spp. is frequently found in patients with poor glycemic control, and it was already shown that increased salivary glucose levels lead to increased oral *Candida* carriage (Al-Maskari et al. 2011, Kumar et al. 2014).

This *in vitro* pilot study tries to further define the links between the high level of glucose present in blood and secretions (including saliva) of diabetic patients, and the development of candidiasis in these categories of patients, by combining our findings with references from the literature. The purpose of this study is to assess the growth rate of CA in the presence of different concentrations of glucose and fructose, two of the main physiopathologically and nutritionally relevant sugars in diabetic patients, in order to obtain a better understanding of the nutrient acquisition strategy and its possible relation to the hyperglycemic status of diabetic patients. The *in vitro* study of *Candida* behavior in the presence of glucose is an attempt to recreate one of the conditions this fungus meets in a diabetic organism. Furthermore, we studied CA behavior in fructose environment, in order to obtain a better understanding of the nutrient acquisition strategy and implication of this glucose substituent from diabetic food products upon the *Candida* replication rate.

We hypothesize that fructose, a sugar that is generally metabolised slower than glucose, will negatively affect CA growth. Standard plate count (SPC) and optical density (OD) measurements are commonly used methods for determining microbial growth (Biesta-Peters et al. 2010). Our previous experiments (unpublished data) showed that SPC is not a reliable method for following CA growth rate, because of high variation between replicates. In order to achieve more consistent results, we assessed the yeast cell population dynamics using the Apogee A50-Micro flow-cytometer (Apogee Flow Systems, UK), specially designed for counting small biological particles, due to its better light scatter performance (Foladori et al. 2010).

## MATERIALS AND METHODS

A 0.5 McFarland suspension (approximately 1-3 x 10^6^ yeast cells/mL) was created from fresh, 24-hour culture of *C. albicans* (ATCC 10231) in saline solution. The strain is part of the microbe collection of the Microbiology Department of the University of Medicine and Pharmacy of Tirgu Mures, Romania. After thawing an aliquot from -80ºC freezer, the strain was subcultured twice on Sabouraud dextrose agar (Becton, Dickinson and Company, Sparks, USA) at 35ºC, 24 h before each experiment. Ten microliters of the 0.5 McFarland suspension were inoculated in 990 µL of sterile nutrient broth (Oxoid, UK). Pure glucose or fructose powder was added in order to obtain stock solutions of 1000 mg% w/v. Lower concentrations of sugars (500 mg%, 250 mg% and 100 mg% w/v, respectively) were prepared by diluting appropriate volumes of the stock solution in nutrient broth. One sample tube with no added sugar served as growth control. Each experiment was made in triplicate. All the samples were incubated in a shaking mixer at 37ºC, in normal atmosphere, for a total of 9 h. At starting time (H0) and every 3 h (H1 = 3 h; H2 = 6 h; H3 = 9 h), the number of yeast cells was assessed by flow-cytometry using Apogee A50-Micro flow-cytometer (Apogee Flow Systems, UK), by counting the number of events in 10 µL of each fungal suspension in a gating area on LALS/SALS scatterplot cytogram that corresponds to the yeast cells population. The obtained values were adjusted to obtain the number of colony forming units/mL (CFU/mL). Each sample was shortly but thoroughly vortexed three times before aspiration, in order to disperse and evenly spread the yeast cells. For a better accuracy, the tests were conducted in triplicate (three tubes for each sample). Because of the complexity of time requirements of this experiment, the glucose and fructose were assessed in two different days, without major variation regarding the environmental and processing conditions.

The CFU/mL values for all samples were normalised against the values obtained at H0 and expressed as Δ-Index, which compensated the errors regarding inoculum and sample preparation (the CFU number at starting point H0). The second normalisation was performed between the Δ-Index of the samples and Δ-Index of the controls (CFU/mL values of the samples with no added sugars), and expressed as ΔΔ-Index, which compensated the growth variation due to external or other non-identifiable factors, thus making it a better growth marker. A ΔΔ-Index value higher than one is equivalent to growth enhancement, while a value less than one is equivalent to growth inhibition, compared to control.

Complementary to the normalised indices, we assessed the growth rate per hour (r) and the generation time (g) in minutes, which were calculated using the absolute numbers of CFU/mL read at H3 compared to H0, according to the following formula:


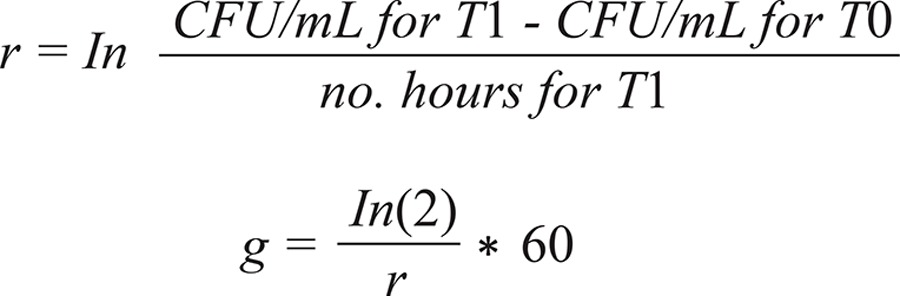


*Explanation: r - growth rate; T1 - time of sample reading (in hours); T0 - initial time (0 hours reading)*

All the statistical evaluations were performed in spreadsheet software and GraphPad InStat3. The significance threshold was considered for a p-value of less than 0.05.

## RESULTS

**Fig. 1:**
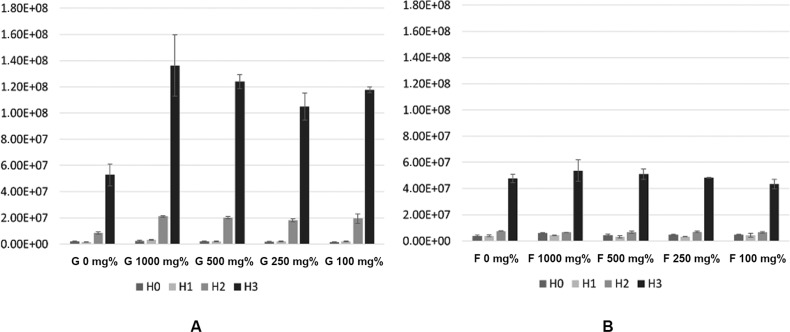
: differences in the number of CFU/mL (mean values) after 9 h of incubation in presence of glucose (A) and fructose (B).

Presence of glucose and fructose presented opposite effects on the growth rate of CA. Overall, glucose had a stimulatory effect while fructose an inhibitory effect on the growth of CA after 9 h of incubation (Fig. 1). In absolute values, all samples presented an exponential, significant growth (p < 0.005). The first 3 h were part of the lag phase, when the number of CFU was constant or decreasing. At H2, after 6 h of incubation, the yeast population was less than double in the case of fructose (Δ-Index between 1.14 and 1.98) or up to 12 folds increased in the case of glucose (Δ-Index between 8.86 and 12.11).

**Fig. 2:**
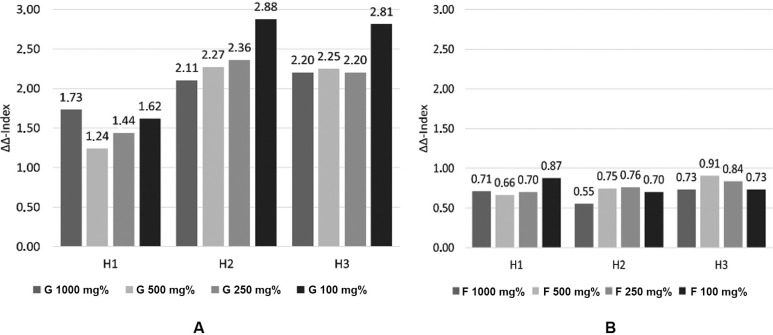
: normalised indices for *Candida albicans* growth in presence of different concentrations of glucose (A) or fructose (B).

**Fig. 3:**
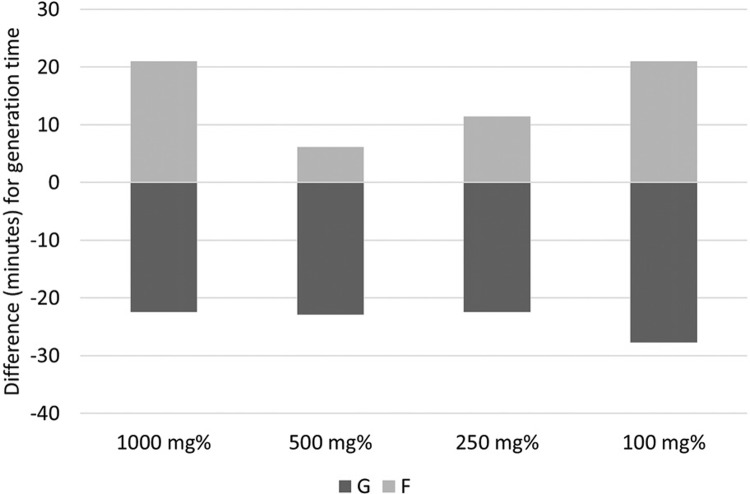
: alteration of generation time in presence of glucose (a) and fructose (b).

The high concentration of glucose (1000 mg%) provided a boost effect on the growth rate of CA as read after 3 h of incubation (H1) but this effect almost leveled during the last 6 h. Instead, glucose 100 mg% presented a milder but sustained effect after the 9 h of incubation, this concentration being the most potent simulator of CA growth rate. Fructose presented an inhibitory effect on CA growth, regardless of its concentration (Fig. 2). The growth rate is directly related to the generation time. Glucose presence decreased the generation time with more than 20 min, while fructose presence increased it with approximatively 15 min. In absolute values, the growth rate/hour ratio was higher in glucose presence (r = 0.45-0.48) than in fructose presence (r = 0.25-0.27). Generation time varied between 87-92 min for glucose and 154-166 min for fructose (Fig. 3).

**Fig. 4:**
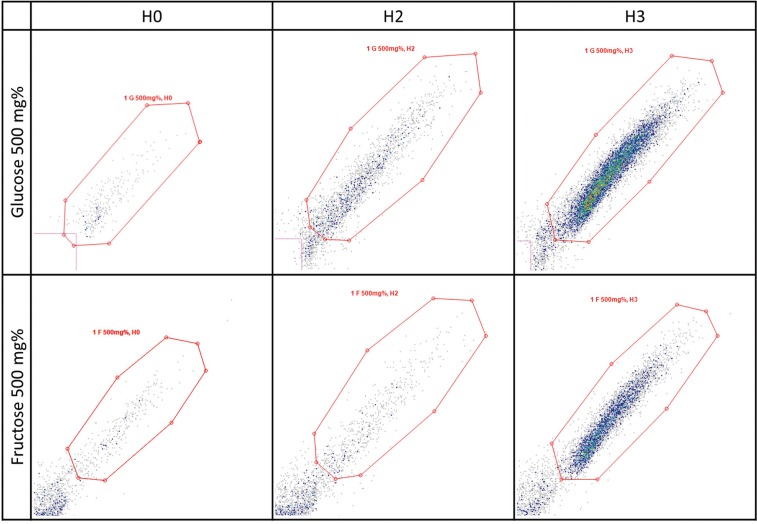
: images of flow-cytometry assay: *Candida albicans* growth in presence of glucose and fructose 500 mg% at three time-points.

The flow-cytometry showed a yeast population that was visible at H0, with no significantly different morphology at H1, which started to be better defined but still largely spread at H2, and condensed and well defined after 9 h of incubation (H3). A second population made of cell debris and probably particles from the culture medium was noticed in all samples, and ignored. These trends were noticed for both sugars. For glucose, even in the concentration that least stimulated the growth rate of CA, the cell population was visibly enhanced at H2 and H3, compared with the corresponding scatter plots of fructose. The small yeast cells (young CA cells) are visible in presence of glucose in the lower-left corner of the gating area at H2 (Fig. 4).

## DISCUSSION

Yeasts and fungi are frequently reported as responsible of severe conditions affecting the cervical and facial areas of patients suffering from immune disorders and diabetics (Giudice et al. 2016), and *Candida* spp. are the most common opportunistic fungal pathogens, CA accounting for more than 50% of the cases (Minea et al. 2015).

The candidiasis can be easily recognised, even if other clinical pictures could simulate the same aspect, as some syndromic conditions (Marrelli et al. 2012), some oncological lesions (Santacroce et al. 2005, Petrovan et al. 2017) or aggressive bacterial infections in immunocompromised patients or as results of infections from foreign bodies (Inchingolo et al. 2011).

The pathogenicity mechanisms of CA are limited compared to those present in bacteria, making this yeast a commensal and opportunistic pathogen that is present in the oral cavity in about 75% of the population, and active in case of host-related disequilibrium such as bacterial disturbance, immune disorders or local pathologies (Jenkinson & Douglas 2002). Hyphal forms, with high invasiveness, blastospores with high dissemination capacity, adhesins, invasins, biofilm formation, environmental factors sensing and metabolic adaptation are part of the pathogenicity factors in CA (Mayer et al. 2013). The metabolic adaptation can have a key role in diabetic patients where glucose levels are higher than in non-diabetic populations, with implication in local or disseminated candidiasis.

Sugars represent potential nutritional sources for yeasts, at least *in vitro*; glucose, fructose and galactose are physiologically relevant in humans. Nevertheless, these sugars seem not to be the single nutrient sources, as lipids, proteins and amino acids are valuable alternative sources, especially in tissues with low glucose concentration (Mayer et al. 2013). Glucose and fructose enhance CA virulence by stimulating the transition from blastospores to hyphae *in vivo* (Sabina & Brown 2009). Glucose concentration is higher but variable in the oral cavity (due to food habits and oral hygiene) than in blood and it seems not to have a primordial role for the burden of *Candida* in oral mucosa, but to enhance the burden of *Candida* in systemic infections (Ene et al. 2012). Nevertheless, in diabetic patients, unlike in non-diabetic ones, permeability of the basement membrane of salivary glands is affected, leading to glucose “leakage” into the saliva (Kumar et al. 2014). This explains why diabetic patients present high rates of oral candidiasis.

The growth boost in presence of 1000 mg% glucose was noticed in the first 3 h of incubation, which partly correspond to the lag phase. This can be explained by the nutritional source abundance, which provided acute energy for CA cells, but had a less favorable osmotic effect in time. Instead, a more convenient concentration of glucose (100 mg%) provided a better environment for long-time CA growth. We did not assess the effect of lower concentrations of glucose on CA growth, but we can suspect that it may similarly stimulate it. This hypothesis is supported by several studies showing that in non-controlled diabetic patients, where the salivary glucose level is higher than 12-13 mg/dL, oral *Candida* carriage is more common (Kumar et al. 2014, Satish et al. 2014). Other studies identified patients with salivary glucose levels of 25-30 mg/dL that also correlated with high *Candida* carriage (Balan et al. 2015). Moreover, the salivary glucose level remains high for up to 2 h after food and/or sugar intake and the recovery rate to normal levels is slower in diabetic than in non-diabetic subjects (Jurysta et al. 2009). The food type also plays an important role in sugar clearance rate (Nirmala et al. 2016), thus high levels of salivary glucose are indeed achievable and may favor *Candida* colonisation.

Previous studies showed that fructose is metabolised more slowly than glucose by CA (Vidotto et al. 1996) and our results regarding the growth rate in presence of fructose support this. Moreover, our results showed that fructose inhibits the replication rate in CA. This may not be true in other osmotolerant and fructophilic species of *Candida*, which present complex sugar transport systems, including fructose-specific ones (Lee et al. 2014). In these cases, growth in presence of fructose may have a completely different trend compared to that of *C. albicans*, as in the latter there is either a metabolic interaction between fructose and the sugar metabolism or a complete lack of fructose transportation systems. This hypothesis needs further studies in order to establish the fructose metabolism within *C. albicans*. Given the fact that fructose improves long-term glycemic control (Cozma et al. 2012), thus being frequently used as a common sweetener by diabetic patients, it may also provide a certain degree of protection against CA infections. On this hypotesis, we have to mention a potential drawback of excessive fructose consumption, as described by DiNicolantonio et al. (2015): *Added fructose in particular (eg., as a constituent of added sucrose or as the main component of high-fructose sweeteners) may pose the greatest problem for incident diabetes, diabetes-related metabolic abnormalities, and cardiovascular risk*, as the isocaloric exchange of other sugars with fructose leads to increased insulin resistance and glucose levels (Tappy & Lê 2015).

We know that the risk of infection with *Candida* reported for the patients with diabetes mellitus has a multifactorial cause. In addition to the diabetic state, high sugar intake, bad eating habits or poor oral hygiene are factors that contribute to high levels of salivary glucose, and are favoring factors for oral candidiasis. Nevertheless, in this context, our study provides further proof regarding the direct link between glucose concentration and candidiasis.

In addition, our results provide information about the fungal fitness abilities in a fructose-rich environment, which raises the question whether fructose diet is a protective factor against oral candidiasis. Fructose intake, as part of diet food (sugar replacement agent) in diabetes, may play a protective role against oral candidiasis in these patients. PubMed search for the terms (“candida/metabolism”) AND “fructose” revealed 40 results, all of them referring to other *Candida* species than *albicans*. This pilot study brings new information on *C. albicans* metabolism related to fructose and to fructose intake that is especially met in diabetic patients. Previous studies showed that diabetes can be conveniently induced in rats (Scridon et al. 2015), so these experimental models will be the target of future *in vivo* experiments regarding the growth rate and infectious potential of CA. Having such consideration in mind, this study is aimed to highlight the importance of the proper clinical approach to the diabetic patients, also from the point of view of the oral care: a deep knowledge of the oral condition is important to reduce the systemic complications of some pathogenetic mechanisms (Santacroce et al. 2010).

In conclusion, *in-vitro* analysis of CA growth rate by flow-cytometry is a new alternative for other commonly used methods, such as optical density measurement or standard plate count, which allows the differentiation of yeast cell populations. Our results showed that there is a positive correlation between the number of CA cells and the hyperglycemic environment, thus providing further evidence on the high incidence of CA infections in patients with poor glucose management. Additionally, we showed that fructose increases the generation time of CA, thus inhibiting its growth rate. Although there is a proven interdependence between the fructose concentration and CA growth rate, the connection between the values is still open for debate. This study is a strong contribute to the knowledge of the numerous pathways that influence the oral cavity and its microbiota: it also remarks the importance of the multidisciplinary approach to the oral pathologies (Mangini et al. 2006). In fact, the correlations between the systemic diseases and the oral manifestations have many links, actually not fully understood, thus, worthy of further investigations by the scientific community (Santacroce et al. 2008).
